# Conditional Deletion of Cytochrome P450 Reductase in Osteoprogenitor Cells Affects Long Bone and Skull Development in Mice Recapitulating Antley-Bixler Syndrome: Role of a Redox Enzyme in Development

**DOI:** 10.1371/journal.pone.0075638

**Published:** 2013-09-25

**Authors:** Satya P. Panda, Anyonya R. Guntur, Srikanth R. Polusani, Roberto J. Fajardo, Peter T. Gakunga, Linda J. Roman, Bettie Sue Masters

**Affiliations:** 1 Department of Biochemistry, University of Texas Health Science Center, San Antonio, Texas, United States of America; 2 Department of Orthopedics, University of Texas Health Science Center, San Antonio, Texas, United States of America; 3 Department of Developmental Dentistry, Division of Orthodontics, University of Texas Health Science Center, San Antonio, Texas, United States of America; National University of Singapore, Singapore

## Abstract

NADPH-cytochrome P450 oxidoreductase (POR) is the primary electron donor for cytochromes P450, dehydrocholesterol reductase, heme oxygenase, and squalene monooxygenase. Human patients with specific mutations in POR exhibit severe developmental malformations including disordered steroidogenesis, sexual ambiguities and various bone defects, similar to those seen in patients with Antley-Bixler syndrome (ABS). To probe the role of POR during bone development, we generated a conditional knockout mouse (CKO) by cross breeding *Por* ^*lox/lox*^ and *Dermo1 Cre* mice. CKO mice were smaller than their littermate controls and exhibited significant craniofacial and long bone abnormalities. Differential staining of the CKO mice skull bases shows premature fusion of the sphenooccipital and basioccipital-exoccipital synchondroses. Class III malocclusion was noted in adult knockout mice with an unusual overgrowth of the lower incisors. Shorter long bones were observed along with a reduction in the bone volume fraction, measured by microCT, in the *Por*-deleted mice compared to age- and sex-matched littermate controls. Concerted up- or down-regulation of proteins in the FGF signaling pathway observed by immunohistochemistry in the tibia samples of CKO mice compared to wild type controls shows a decrease in the FGF signaling pathway. To our knowledge, this is the first report of a mouse model that recapitulates both skull and long bone defects upon *Por* deletion, offering an approach to study the sequelae of POR mutations. This unique model demonstrates that P450 metabolism in bone itself is potentially important for proper bone development, and that an apparent link exists between the POR and FGF signaling pathways, begging the question of how an oxidation-reduction flavoprotein affects developmental and cellular signaling processes.

## Introduction

NADPH-cytochrome P450 oxidoreductase (POR) is the primary electron donor for various endoplasmic reticulum (ER) resident proteins such as cytochromes P450 (CYPs), heme oxygenases (HOs), squalene monoxygenase, and fatty acid elongase [1]. CYPs catalyze monooxygenation reactions of many endo- and xenobiotics. Among CYP-mediated reactions, CYP26 metabolizes retinoic acid (RA), a known teratogen, the metabolites of which are critical during embryonic development [2,3]. CYP51 (lanosterol 14α-demethylase) catalyzes the demethylation of lanosterol and 24,25-dihydrolanosterol in the cholesterol biosynthesis pathway, utilizing electrons from POR [4]. CYP2R1 in the liver converts vitamin D3 to 25-OH-vitamin D3, the first step in the formation of activated dihydroxyvitamin D_3_ [5]. CYP17, which catalyzes both 17α hydroxylase and 17, 20-lyase reactions, is crucial for sexual development in the fetus and at puberty [6]. CYP19, known as aromatase, catalyzes the aromatization of androgens to estrogens [7]. Heme oxygenase, a key regulator of intracellular heme pools that converts toxic heme into biliverdin, ferrous iron (Fe^+ 2^) and carbon monoxide [8], has also been shown to utilize electrons from POR for its activity [9]. Since POR is the only known electron donor for the CYPs and heme oxygenases, any alteration in its activity can be expected to have pleiotropic effects. Indeed, certain mutations in the *POR* gene in the human population are accompanied with defective skeletal development similar to that of the previously described Antley-Bixler syndrome (ABS) [10], as well as aberrant steroid metabolism and ambiguous genitalia [11]. In addition, mutations in *POR* leading to bone deformities have been shown to segregate from fibroblast growth factor receptor II (FGFRII) mutations associated with ABS [12].

The vital role of POR in development was demonstrated by the observation that complete deletion of POR was embryonically lethal in mice and resulted in developmental defects involving limb bud development and vascularization [2,13]. Deletion of the *Por* gene in liver, heart, intestine, lung, and neurons, using tissue-specific *Cre* mice, by Drs. X. Ding’s [14,15,16,17,18,19] and R. Wolf’s groups [3] has permitted the understanding of the function of POR in these tissues. The appearance of craniofacial and long bone defects in human patients with POR deficiency led us to examine the effect(s) of specific deletion of the *Por* gene in the bones of mice. Tissue-specific deletion of the *Por* gene using Cre/Lox technology was utilized to interpret the specific role of the reductase in various tissues and it also precluded the embryonic lethality of the complete *Por* gene knockout in mice. The only previously published work was by Schmidt et al. (2009) [20],, in which the role of POR deletion in limb development was examined by generating conditional deletion mice utilizing *Prx1 Cre* and *Por* ^*lox/lox*^ mice.

In the present study, we have focused on understanding the role of POR in bone development by deleting *Por* in the mesenchyme by crossing *Dermo1 Cre* (*Twist2*) [21] and *Por* ^*lox/lox*^ [14] mice. In the *Dermo1 Cre* mice, Cre recombinase is expressed in the mesenchyme, including both chondrocyte and osteoblast cell lineages, determining that the targeted deletion of POR will occur in both of these cell types. *Dermo1 Cre* mice have been used for conditional deletion of genes in osteoprogenitor cells to understand the role played by these genes during skeletogenesis, including skull development and signaling events during development [22,23,24,25]. Use of *Dermo1 Cre* for the deletion of *Por* allowed us to evaluate its possible role in skull development, whereas the previous work that used *Prx1 Cre* to delete *Por* failed to target the skull base and thus most likely did not result in any skull defects. The *Prx1 Cre* is shown to be expressed in some of the craniofacial bones, but not in the skull base [26].

Our studies demonstrate that deletion of POR in osteoprogenitor cells reduces the body size of these mice compared to the sex-matched littermate controls. The bone volume fraction of trabecular bone was found to be reduced in these conditional knockout mice, as measured by micro computed tomography (µCT), whereas no significant difference was observed in the cortical bones. These mice were also shown to have dome-shaped skulls with tooth malocclusions (Class III) due to premature fusion of synchondroses in the skull base. To best of our knowledge, this is the first mouse model showing skull deformities upon deletion of *Por* [27] and the only other model with *Por* deletion. The presence of skull deformities has been reported in humans harboring the more severe mutations in the *POR* gene [12]. It is of great interest to delineate the function of POR and various metabolites of the CYP-mediated pathways in the microenvironment of bone during development, especially since the importance of CYP-mediated metabolism in bone has not previously been shown. Furthermore, a well-characterized mouse model of bone-specific deletion of *Por* may be used for discovery of therapeutic intervention in *POR*-deficient patients.

## Materials and Methods

### Ethics Statement

All animal studies have been approved by Institutional Animal Care and Use Committee (IACUC), University of Texas Health Science Center at San Antonio, San Antonio, TX, USA (Protocol id: 07099z). Animals are housed in an Association for Assessment and Accreditation of Laboratory Animal Care, International (AAALAC) accredited facility with full veterinary support. The facility is operated in compliance with the Public Law 89-544 (Animal Welfare Act) and amendments, Public Health Services Policy on Humane Care and Use of Laboratory Animals (PHS Policy), and The Guide for the Care and Use of Laboratory Animals as the basis of operation.

### Generation of conditional knockout mice

*Por* ^*lox/+*^ females were crossed with *Dermo-1* ^*Cre/+*^ *Por* ^*lox/+*^male to generate wild type and conditional knockout mice (*Dermo1* ^*Cre/+*^ *Por* ^*lox/lox*^). Mice genotypes were confirmed by using DNA from tail snips and published primers [21]. Only male mice were used for the studies presented in this report to simplify analysis and to avoid any complication due to gender-specific variations.

### Primary osteoblast isolation

Primary osteoblasts were isolated from calvariae of three-day-old pups as described by Guntur et al. [25] and Yeh et al. [28]. In short, collected calvariae were scraped from any attached tissues and digested with shaking in a mixture of trypsin (0.05%) and collagenase II (0.2%) in the 37°C CO_2_-humidified chamber. Cells were collected every 15 minutes post-digestion. Cells from five such digestions were collected after discarding the first two digestions to minimize contamination of other cells. **Western blotting and qPCR**: POR protein expression in wild type and CKO was determined by Western blotting using cell extracts from isolated primary osteoblasts from mouse calvariae and quantified using ImageJ (http://rsb.info.nih.gov/ij/). Similarly, POR mRNA levels in wild type and CKO were determined using qPCR. Data presented were normalized to the 18S mRNA level.

### Differential staining of whole skeletons

Euthanized mice at different ages were skinned and eviscerated. Bone and cartilage of these mice were differentially stained using Alizarin red and Alcian blue as described before [29].

### Micro Computed Tomography analysis

Micro CT of tibia samples was carried out in the Department of Orthopedic’s Micro CT Core Facility Laboratory at University of Texas Health Science Center at San Antonio under the supervision of Dr. Roberto Fajardo and James Schmitz. Tibial image data were acquired on a SkyScan 1172 desktop micro-CT system using the following acquisition parameters: 59 kV, 167 mA, 0.7 ° rotation step, 4 frame average, and a 10 µm nominal isotropic resolution for the 16-day and 16-week old groups. A 6 µm nominal isotropic resolution was used for the 1-day old group.

### Bone Mineralization Assay

Primary osteoblasts isolated from calvariae were counted and cultured in 12-well culture dishes. Ascorbic acid (50 µg/ml) and β-glycerophosphate (10 mM) are added once the cells reached 100% confluency for 2 or 3 wk. Once mineralization was observed, wells were washed three times with TBS, fixed with 10% neutral formalin solution for 5 min, and rinsed with deionized water. Von Kossa staining was done by the addition of 5% silver nitrate solution, and exposing wells to UV light for 1 h [30]. The plates were rinsed with deionized water, and the residual silver nitrate was neutralized with 5% sodium thiosulphate. Mineralization was quantified by calculating the surface area (in cm^2^) covered by the black stain in each well using ImageJ (http://rsb.info.nih.gov/ij/).

### Immunohistochemistry

Paraffin–embedded, decalcified long bones were sectioned at 6-micron thickness for immunohistochemistry. Citrate buffer antigen retrieval was carried out for these sections followed by 1-hour blocking at room temperature and overnight incubation in 1° antibody at 4°C. Sections incubated with 1° antibody were developed using Tyramide® amplification kit (PerkinElmer, Waltham, MA) using vendor supplied protocol. The average intensity (n=3) was calculated using Metamorph Software (Molecular Devices, Downingtown, PA).

### Statistical Analysis

Data and standard errors presented were average of three independent experiments with littermate and sex-matched controls. Statistical significance was calculated using Student’s t-test.

## Results and Discussion

### Conditional deletion of POR in mouse mesenchymal cells

**Figure 1:**
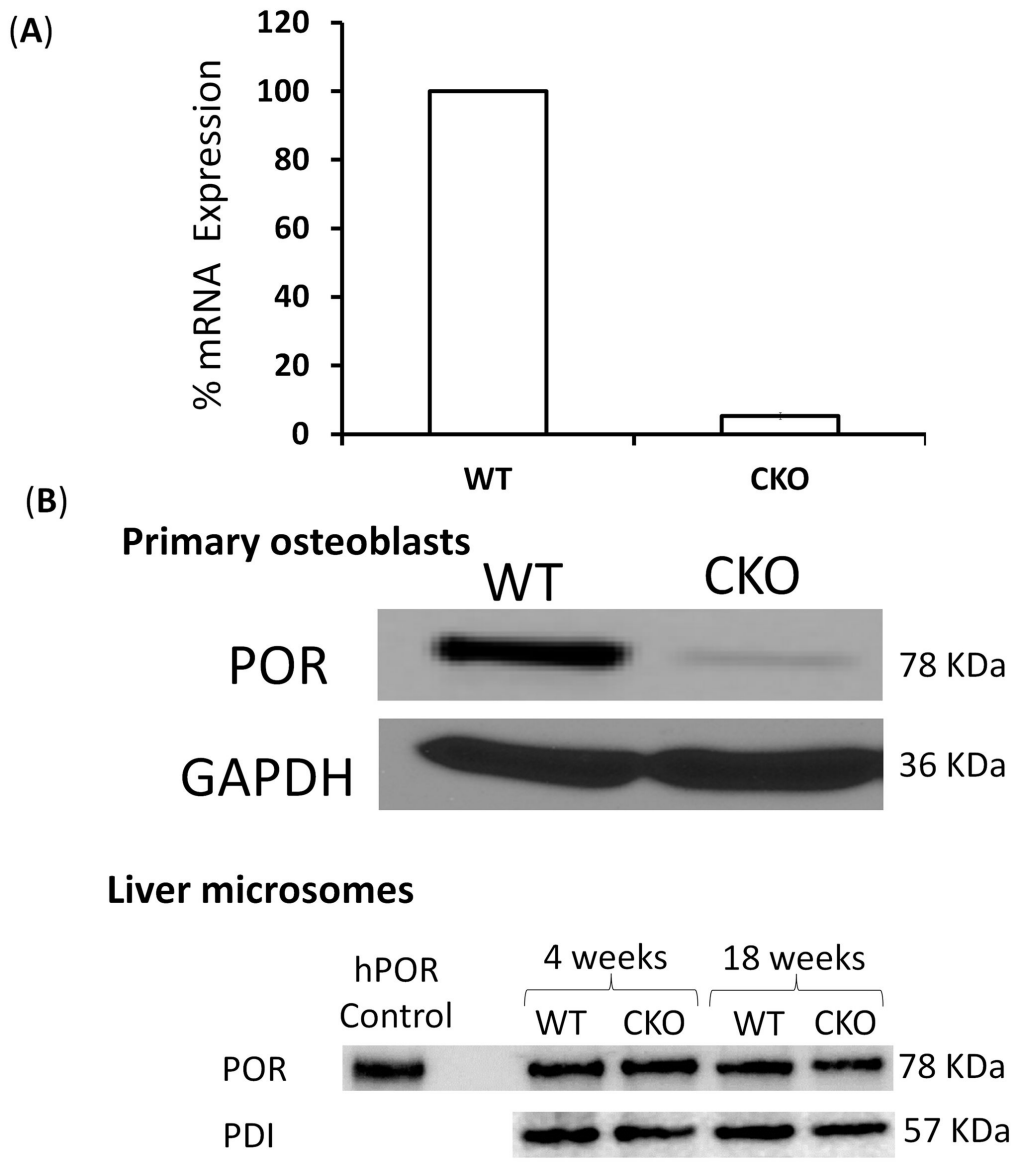
POR expression in wild type vs. **CKO mice littermates**. Expression of POR in the primary osteoblasts isolated from wild type and conditional knockout mice was examined at the RNA level using quantitative PCR (n=2) (A) and at the protein level with immunoblotting (B). POR expression in liver was compared between wild type and CKO mice at 4 and 18 weeks of age (B). Protein disulfide isomerase was used as a control for protein quantity ran in each lane. Recombinant human POR was used as positive control.

POR was deleted from bone by crossing *Dermo1 Cre* mice with *Por* ^*lox/lox*^ mice [21]. Publications from other laboratories using *Rosa26* reporter mice have shown that during endochondral ossification, *Dermo1 Cre* expression was observed in condensed mesenchyme and later in chondrocytes in growth plate cartilage and in osteoblasts in perichondrium, periosteum and endosteum. The Cre activity is also detected in the osteogenic front during intramembranous ossification of developing sutures. Detection of Cre activity in cell types from *Dermo1 Cre* and *Rosa26* mice cross breeds suggests that POR is deleted from chondrocytes and osteoblasts by the action of Cre [21]. Deletion of POR from bone cells was confirmed by both qPCR and western blotting. Analysis of primary osteoblasts isolated from *Por*-Bone CKO mice calvaria showed decreased expression of POR at both gene and protein levels as compared to the wild type littermate controls (Figure **1**). Quantitative PCR analysis using cells from two independent litters (n=2), each ran in triplicate, showed a decrease of 94.6±1.99% at the gene level, which is reflected as a decrease of 91.5±4.4% at the protein level as analyzed by Western blot. To ensure that any phenotype observed in these CKO mice was not a consequence of lower POR levels in the liver, the major organ for cytochrome P450-mediated metabolism, liver microsomes were also probed for POR protein levels (Figure **1B**). The CKO mice demonstrate a level of POR in liver comparable to that of wild-type. Protein disulfide isomerase (PDI) was used as a loading control for microsomal POR.

### Phenotypic observation of the CKO mice

**Figure 2:**
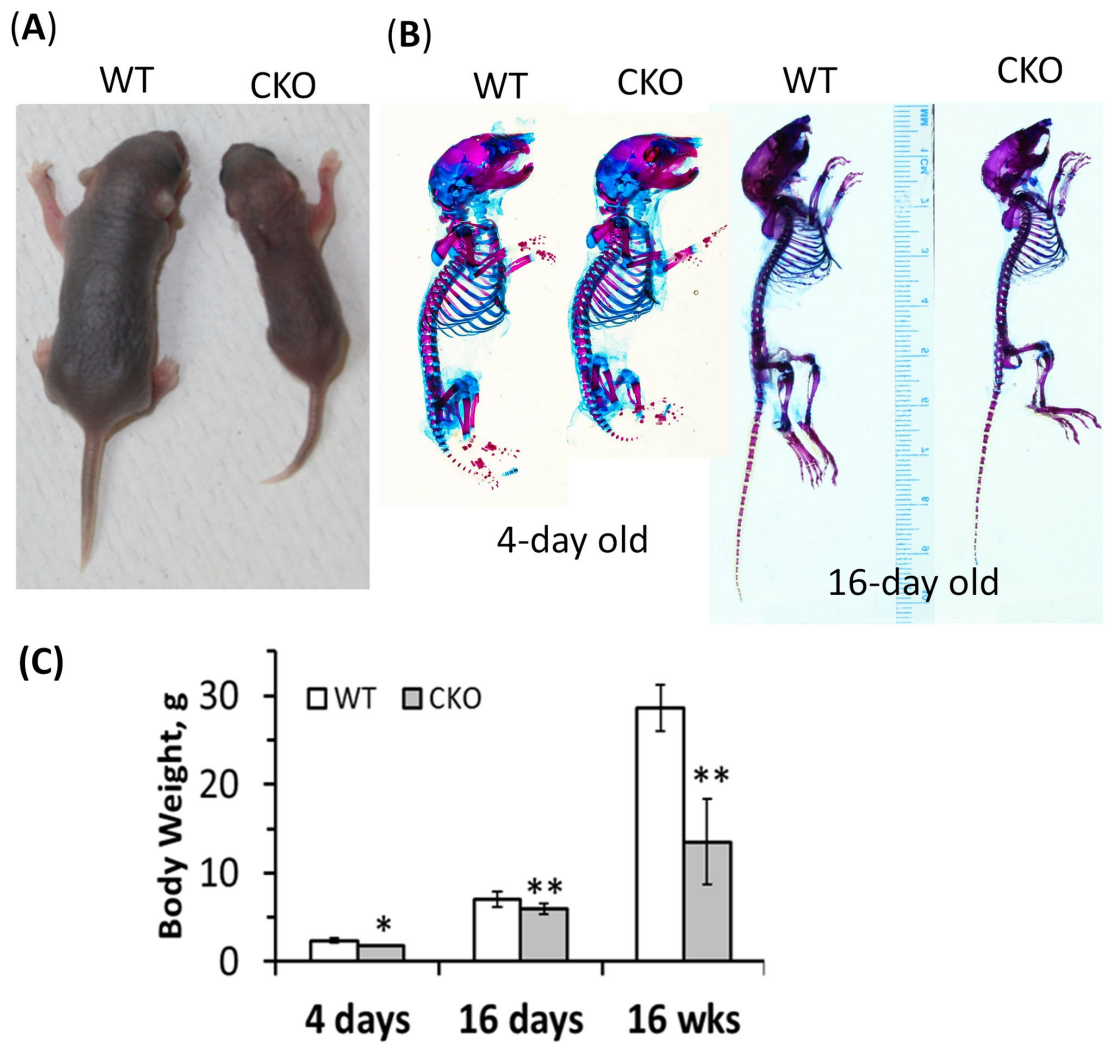
Body size/weight differences in littermates. (**A**) Photograph of 5-day-old mice; (**B**) differential stain of 4- and 16-day-old mice (images not taken to scale, precluding comparisons between age groups); and (**C**) compiled body weights of 4-, 16- and 16-week-old wild type (open bars) and CKO (solid bars) mice (*, p < 0.005 and **, p < 0.001).

The CKO mice were smaller in size compared to their wild type littermates. The differences in body weight and size were visible very early (Figure **2**). Whole animals differentially stained with Alizarin red and Alcian blue also showed a reduction in skeletal size. Body weights of litters containing CKO mice were measured at different ages (4 days (n=3), 16 days (n=10) and 16 weeks (n=5) of age) and the CKO body weight was found to be lower in all cases (Figure **2**). The larger difference observed in the older animals can possibly be attributed to the decreased food intake by CKOs showing a tooth malocclusion, which was not the case for every litter (**Table S1**). This is reflected in the increased variability and much lower body weight seen in 16-week CKO mice (Figure **2B**). The tooth malocclusion is discussed in detail later. Embryos from complete *Por* knockout mice, which are lethal by embryonic day 13.5 (E13.5) or E10.5, depending on the design of the deletion construct, showed a “general retardation of development”, along with defects in neural tube, cardiac, eye, and limb bud development or absence of limb bud [2,13]. However, mouse models with *decreased Por* gene expression (74% to 95% decrease) were not embryonically lethal, but did show overall developmental retardation [15]. The conditional deletion of *Por* targeted at limb bud mesenchyme using *Prx1 Cre* led to defects in both patterning and skeletogenesis, as well as smaller body size in the mice [20], but tissue-specific deletion of *Por* in liver, kidney, lung, heart and neurons apparently showed no effect on the body size. Complete ablation of CYP51, which also led to embryonic lethality in mice due to cardiac malformation, resulted in embryos showing abnormal skeletal development, as well as a reduction in embryo size [31], indicating a role for sterol biosynthesis in skeletal development.

### Skeletal abnormalities in *Por*-Bone CKO mice

**Figure 3:**
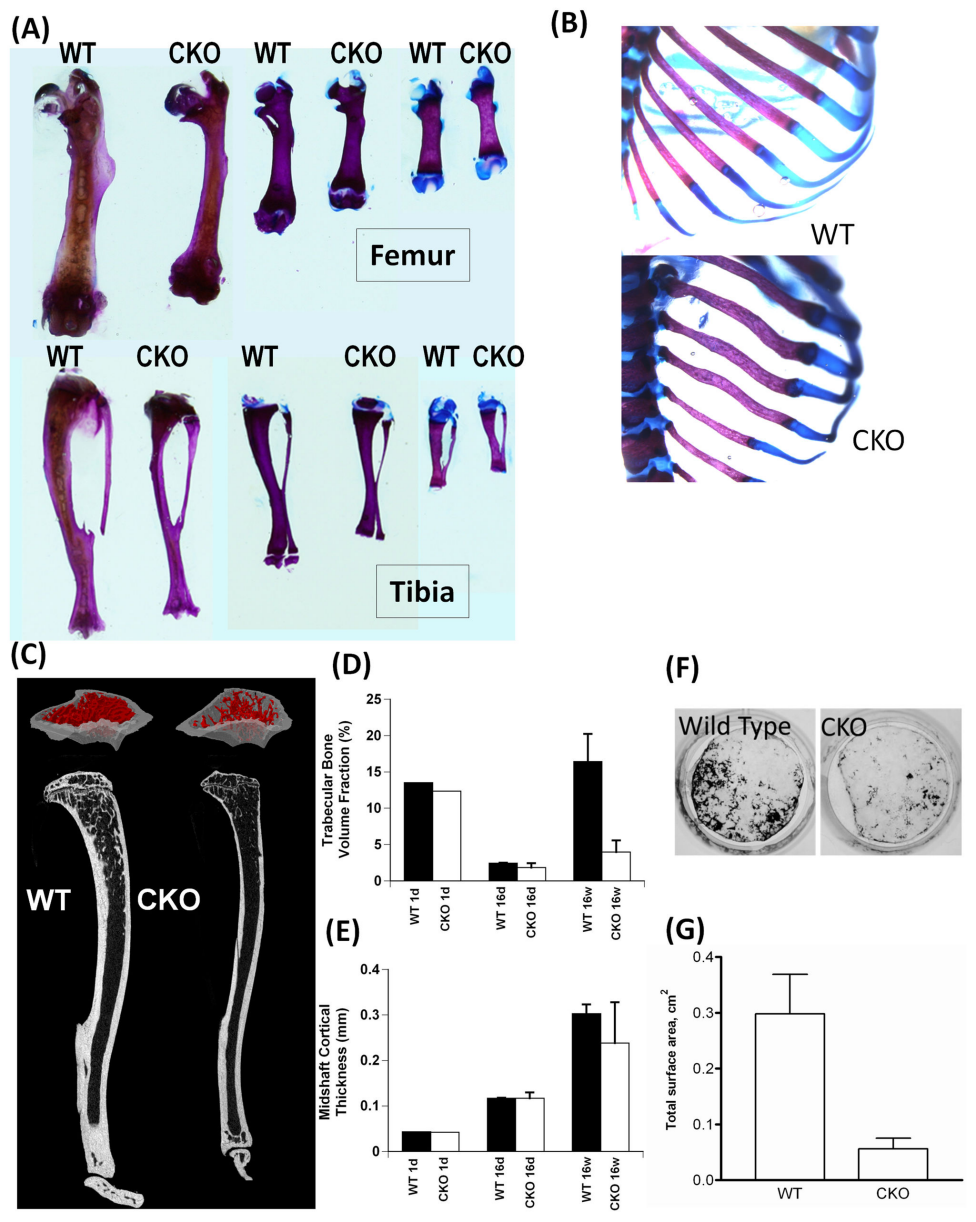
Images of differentially stained femurs, tibias and rib cage of 4-day-old mice. Whole animal littermate pups are differentially stained using Alizarin red and Alcian blue and the images of (A) femur and tibia were taken after disarticulating them from the whole stained animal. This represent observations from n=3 for 4 days, n=4 for 16 weeks and n=7 for 16 weeks old animals. (B) The rib cage image of 4-day old pup was taken using a stereoscope. The rib cage images are not taken to scale. (C) Mid-substance grey-scale and three-dimensional views of proximal tibia trabecular bone in 16-week-old mice show reduced bone volume fraction (D). At 16 weeks, cortical bone thickness in the midshaft is also lower in the CKO mice (E), but little difference is seen in cortical bone at earlier time points. (F) Bone mineralization using primary calvarial osteoblasts: von Kossa staining was performed with primary osteoblasts isolated from calvaria of wild type and CKO littermate mice pups (3-day-old) grown in differentiation media. (G) The surface area of mineralized matrix was quantified using ImageJ from three independent mineralized experiments (n=2).

All *Por*-Bone CKO mice showed reduced body size. Examination of differentially stained appendicular skeleton showed shortened femur and tibia (Figure **3A**). Other than the length, no apparent structural abnormality was evident in either femur or tibia. The difference in length is clearly seen as early as 4 days of age, and the defect was persistent, as seen in the 16-day- and 16-week-old femurs and tibias. In the *Por*-Bone CKO mice, although the limbs appeared normal, albeit shorter, the ribs were jagged and the bone-cartilage joints were abnormal as compared to the wild type (Figure **3B**). A recent publication from our group demonstrated that deletion of POR in osteoblast cell lines, 2T3 and MG63, as well as in the primary osteoblasts isolated from the calvaria of the presently described *Por*-Bone CKO mice, resulted in decreased Connexin 43 (Cx43) expression and gap junctional intercellular communication (GJIC) [32]. Cx43, a major gap junction protein expressed in osteoblasts, is important during bone development. Complete deletion of Cx43 (Cx43^-/-^) in mice led to death after birth accompanied by many developmental defects [30]. Cx43^-/-^ mice showed delayed ossification of limbs, vertebrae and ribs along with shortened limb bones as well as jagged rib cages [30,33]. These observations suggest that the observed bone defects due to loss of POR may be partially contributed by the lower expression of Cx43. **Micro-architecture of bone**: Wild type and *Por*-Bone CKO trabecular bone micro-architecture were compared using µCT (Figure **3C**). Tibia from *Por*-Bone CKO mice, and littermate controls at three time points, 1-day- (n=1), 16-day- (n=2), and 16-week-old (n=3) were analyzed for three-dimensional trabecular bone parameters at the proximal metaphysis. At each time point, the *Por*-Bone CKO showed indications of reduced trabecular bone formation and this effect became more dramatic with advanced age. Specifically, there was a five-fold difference in trabecular bone volume fraction by 16 weeks of age (Figure **3D**). In addition, the trabecular lattice was comprised of thinner (trabecular thickness) and fewer (trabecular number) trabeculae compared to controls. The reduction in bone formation during development, along with shorter tibiae and dysplasias of the basicranium, is consistent with a malfunction in *endochondral ossification*. Interestingly, the data show a low point in bone volume fraction (BV/TV) at 16 days, which appear to be related to a transition from the presence of thin, numerous primary trabeculae at 1 day to thicker, fewer and mature remodeled trabeculae by 16 wks (Figure **3D**). In contrast, the total cortical bone area and cortical thicknesses are similar between controls and CKO mice (Figure **3E**). **Delayed bone mineralization of primary osteoblasts from *Por*-Bone CKO**: Consistent with decreased bone volume fraction in *Por*-Bone CKO mice, the calvarial osteoblasts also exhibited decreased mineralization potential when grown in osteoblast differentiation media containing ascorbic acid, β-glycerophosphate and bone morphogenetic protein-7 (BMP7), when compared to the wild type (Figure **3F**). Figure 3G shows that the mineralization area in CKO was ~80% less than that of WT (WT 0.3±0.07 cm^2^ vs. CKO 0.06±0.02 cm^2^). Since calvarial osteoblasts from *Por*-Bone CKO mice have decreased Cx43 [32] and both *Por*-Bone CKO and Cx43^-/-^ mice exhibit decreased mineralization potential, it is possible that decreased Cx43 expression in the *Por*-Bone CKO mice might be partially responsible for some of the observed skeletal defects. The *Dermo1 Cre*/*Por* ^*lox/lox*^ mouse knocks POR down in chondrocytes as well as in osteoblasts. The siRNA-mediated knockdown of POR in rat chondrocytes reduced both proliferation and differentiation [34]. These cells exhibited lower cholesterol levels as well as reduced levels of Indian hedgehog (Ihh) protein expression. Exogenous addition of cholesterol or recombinant Ihh protein to the chondrocyte culture medium improved differentiation, suggesting that cholesterol, an essential component of Ihh signaling, is important in the differentiation process [4]. The feeding of a cholesterol-supplemented diet to the dams of *Prx1 Cre/Por* ^*lox/lox*^ was able to rescue, only partially, the soft tissue webbing in the CKO embryo [20], indicating the importance of POR metabolites involved in processes other than sterol metabolism.

### Analysis of skull structure and tooth malocclusion

**Figure 4:**
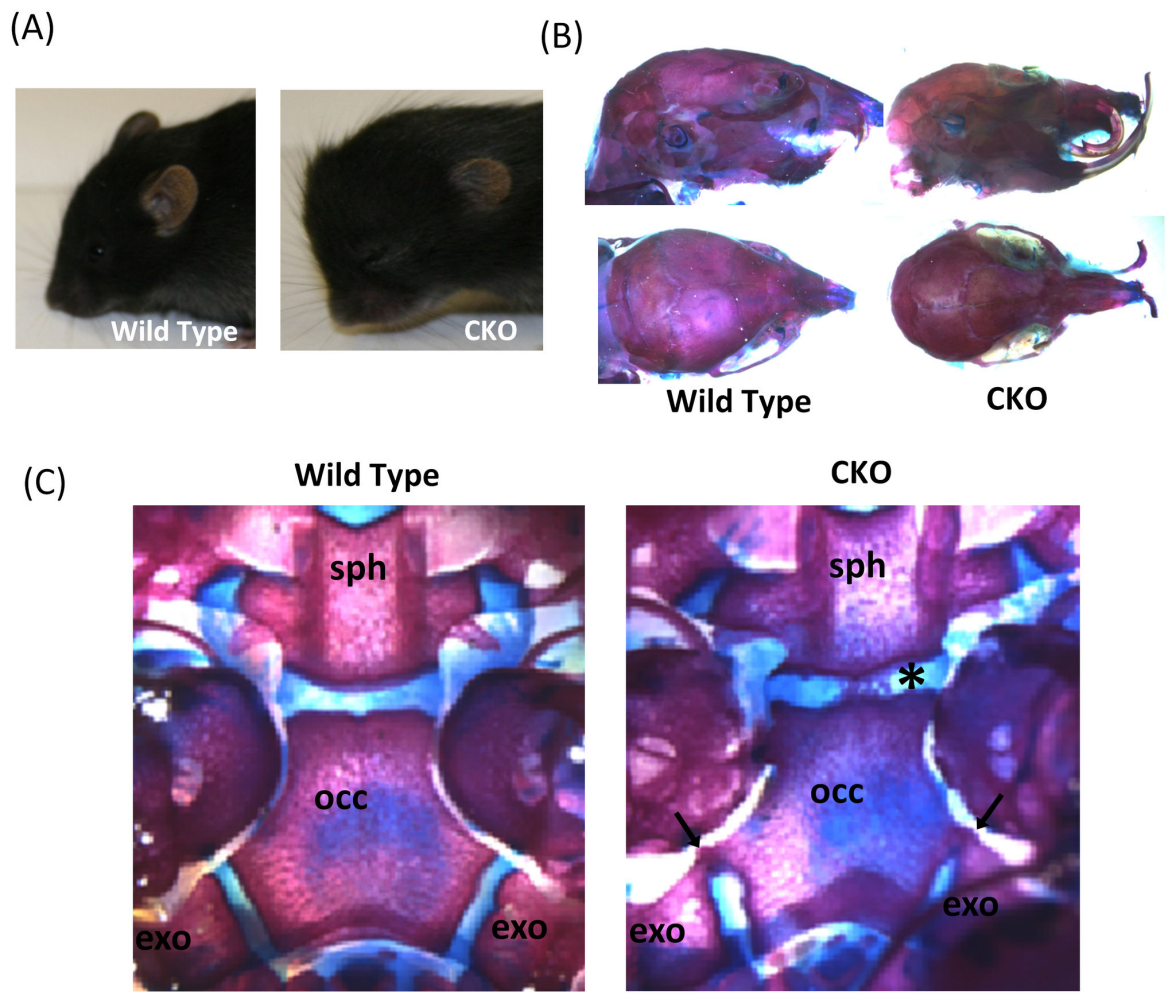
Skull phenotype. (A) Dome-shaped skull present in the case of 16-day-old CKO mouse; (B) dome-shaped skull and tooth malocclusion in 16-wk-old differentially stained skull (C) differentially stained skull base of 4-day old mice showing premature fusion of sphenooccipital synchondroses, SOS, black asterisk, and basioccipital-exoccipital synchondroses, BES, [black arrow], in case of CKO (sph: spheno-occipital bone, occ: occipital bone, exo: exo-occipital bone). These observations were made with n=4 animals in each case.

The *Por*-Bone CKO mice were viable at birth; however, they were smaller than their wild type littermates. This difference in overall body size was replicated in the craniofacial phenotype. The *Por*-Bone CKO mice exhibited an abnormal dome-shaped skull, deficient midface, and a relatively prognathic mandible; indicative of a short cranial base (Figure **4A**). To further examine the craniofacial deficiencies, skeletal preparations and differential staining of 16-week-old *Por*-Bone CKO mice were performed. The data demonstrated significantly shortened maxillary regions, and the lower incisor teeth of the *Por*-Bone CKO mice were positioned anterior to the maxillary incisors, compared to their wild type littermates (Figure **4B**). This combination of a deficient maxilla, and a relatively normal mandible recapitulates a midfacial hypoplasia phenotype, classically defined as a class III facial skeletal type and typifying that observed in patients with POR deficiency. This class III malocclusion became progressively severe between birth and 16 weeks of age and occurred in tandem with the lack of maxillary growth. Due to the malocclusion, the *Por*-Bone CKO mice were unable to feed properly, partially explaining the larger difference in the bodyweights of the longer-lived mice. Differential staining of the cranial bases of 4-day-old mice revealed premature fusion of the cranial base synchondroses in *Por*-Bone CKO mice, compared to their wild type littermates (Figure **4C** **, n=4**). The sphenooccipital synchondroses (SOS) of the *Por*-Bone CKO mice contained bridging calcified elements in the center of the growth plate, while the bony bridging in the basioccipital-exoccipital synchondroses (BES) was toward the edges of the two bones. In normal wild type mice, the cranial base synchondroses are fully established and patent at 4 days of age. This study shows that *Por*-Bone CKO mice display postnatal shortening of the cranial base, which is associated with the formation of bony bridges across the cranial base synchondroses. This reduces the normal growth rate in the cranial base, and may even terminate it.

The craniofacial phenotype observed in the *Por*-Bone CKO mice, especially when combined with the other features described in this paper, are pathognomonic of ABS described in humans, a rare multiple congenital anomaly with a high mortality rate. The distinctive craniofacial malformations include brachycephaly, frontal bossing, midface hypoplasia, depressed nasal bridge and proptosis. These pathological features are associated with disturbance of craniofacial growth due to premature fusion of the cranial base synchondroses. Class III malocclusion of a human patient diagnosed with ABS has been reported [35], although no definitive genetic evidence for ABS or POR mutation was presented in that study. As many severe POR deficiency patients exhibited acute bone deformities similar to those seen in ABS, they were grouped under ABS, but are segregated from FGFR2 mutations, as endocrine effects were also present, and they were shown to be distinctly associated with POR mutations [11,12].

Various genetic factors, such as Fgf2, Bmp2, Runx2, Twist, Tfgβ2 and 3, influence the development of the skull, which includes the cranial vault and the cranial base [36]. FGF signaling during embryonic and bone development, in particular, is a complex process that many laboratories are actively studying. Several mouse models with complete knockout or tissue-specific deletion of FGF receptor genes or replacement of these genes with mutations found in human patients show various bone defects. Conditional deletion of Fgfr2 using *Dermo Cre* mice led to smaller mice and failure in development of several metatarsal joints due to a defect in osteoblast proliferation [21]. A mouse model of Apert syndrome, expressing an activated FGFR2 mutation, showed malocclusion [37]. However, aberrant activation of FGFR2 signaling in mice upon introduction of the Fgfr2IIIC Pro253Arg mutation led to premature fusion of SOS and BES [37] similar to that seen in our POR CKO mice. Fusion of cranial base synchondroses has been reported in transgenic mice expressing the Muenke Syndrome mutation, FGFR3Pro244Arg [38,39]. Deletion of fibroblast growth factor receptor type 2, IIIc isoform (*Fgfr2IIIc*), from mice leads to delayed ossification, loss of growth and synostoses of certain sutures [40]. Knock-in mice with the *Fgfr2c* C342Y mutation, found in Crouzon and Pfeiffer syndromes in humans, showed joint fusions, cleft palate, tracheal and lung defects in the homozygote, and shortened faces accompanied by premature fusion of cranial sutures in the heterozygote [41]. The close resemblance between craniofacial deformities associated with abnormal FGF signaling and severe POR deficiency in humans warrants a close analysis of possible cross- talk between FGF signaling and POR-mediated metabolites.

### Regulation of Bone Development in the Por-Bone CKO mice

**Figure 5:**
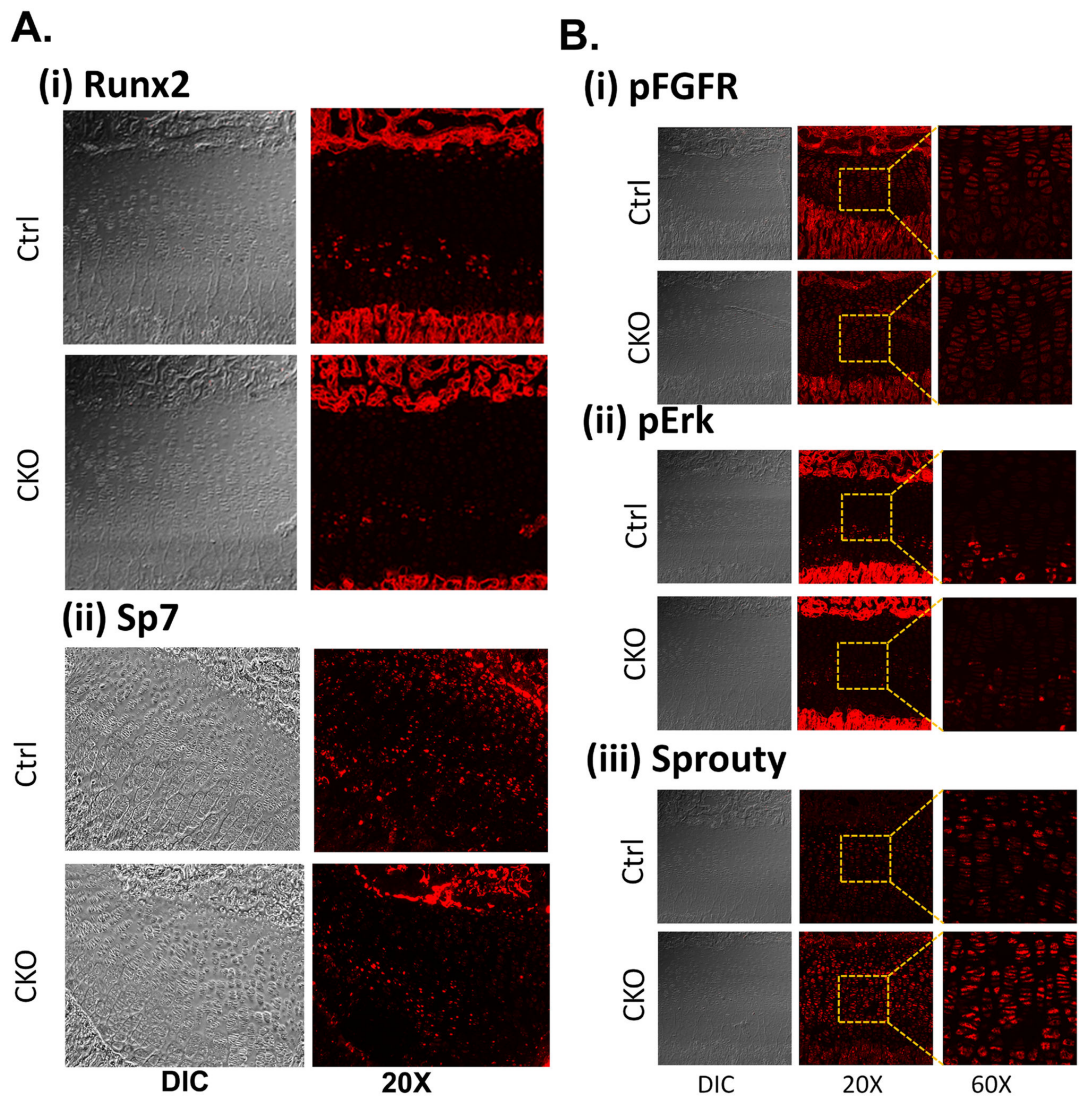
Immunofluorescence of tibia sections of wild type and *Por*-Bone CKO. Paraffin embedded tissue sections from 16-days-old male wild type and Por-Bone CKO mice were stained for (A-i) Runx2, (A-ii) Osx and (B-i) pFGFR, (B-ii) pErk and (B-iii) Sprouty (Spry). The signal is amplified using Tyramide amplification kit®. Images were taken with emphasis on growth plate region with 20X magnification for Runx2 and Osterix along with DIC image. 20X and 60X magnification images were taken for pFGFR, pErk and Spry along with the 20X DIC image. The square outlined in the 20X images shows the area magnified in the 60X images. Each image is representative of n=3 independent staining using three independent tissue samples.

**Figure 6:**
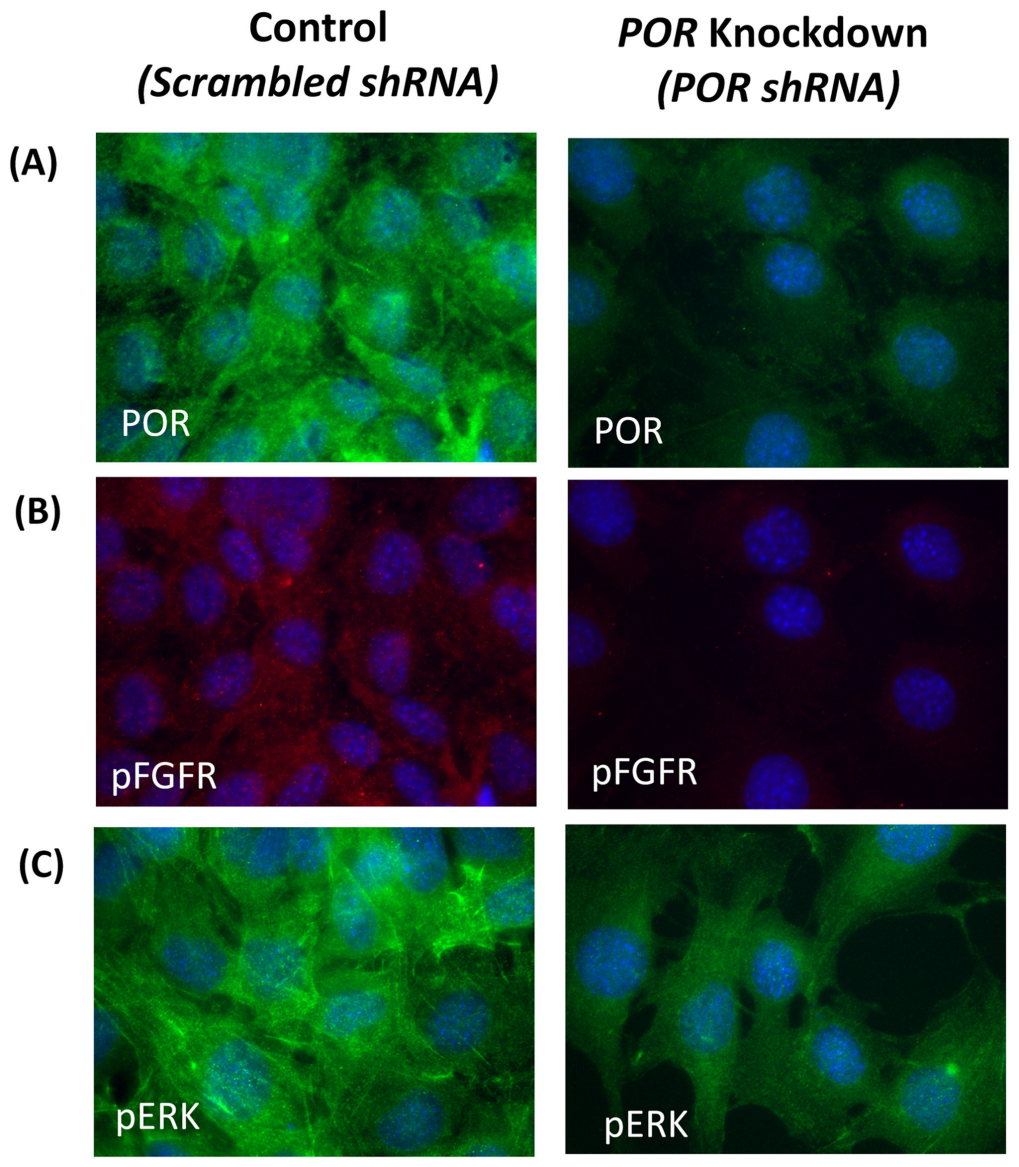
Immunocytochemistry of stably POR knockdown 2T3 cells. Stable clones of shRNA mediated POR knockdown 2T3 cells along with scrambled shRNA clone were stained for (A) POR, (B) pFGFR, and (C) pERK, using antibodies against these proteins and developed using fluorescent-labeled secondary antibodies. DAPI (blue fluorescence) was used for nuclear staining.

Effect on Transcription Factors, Runx2 and Osterix: Among the various transcription factors known to regulate bone development [42,43], two of the most important regulators are Runx2 and Osterix (Osx, also known as Sp7). We probed the POR-cko bones to understand how Runx2 and Osterix expression correlates with the decreased bone volume in *Por*-Bone CKO mice. Runx2, a member of Runt domain transcription factors family, is one of the earliest determinants of both endochondral and intramembranous ossification. Knockout mice of Runx2 completely failed to produce mature osteoblasts suggesting the critical role played by this transcription factor during bone development [44]. Osx, a zinc finger-containing transcription factor acts downstream of Runx2, which is absolutely necessary for osteoblast differentiation [45]. Osx specifically induces osteoblast differentiation and bone formation, Runx2 is involved in both osteoblast differentiation [46]. Immunohistochemical analysis of long bone sections of *Por*-Bone CKO mice for both Runx2 and Osx showed a decrease in expression compared to wild type littermate sex-matched controls (Figure **5A**). The expression of Runx2 decreases by 40% compared to WT as measured by average intensity of the immunohistochemical staining (n=3, Average intensity WT 13.5±2.7 vs. CKO 8±1.4) (Figure **5A** **i**). This suggests that deletion of POR in mesenchymal cells leads to decrease in Runx2 expression. A decreased expression of Osx was also observed in tissue samples from mice collected at ages of 4 days, 16 days and 16 weeks (16 days sample image is shown in the figure, Figure **5A** **ii**). That deletion of POR leads to downregulation of Runx2 and Osx is most likely due to alterations in the concentrations of metabolites formed by Por mediated reactions. Runx2 promotor, P1, which supports osteoblast-specific gene transcription, responds to steroid hormones [47]. FGF Signaling: Fibroblast growth factors (FGFs), which signal through cognate receptors primarily involving a RAS-MAP kinase pathway, which includes ERK1/2, and the PI3K/Akt and the PLCγ pathways [48], are critical during bone development [49] as various autosomal dominant and sporadic mutations of FGFR are present in almost all skeletal disease syndromes and deformities, including Antley-Bixler syndrome. To investigate the possible involvement of POR-mediated metabolism and FGF signaling, proteins downstream of FGF signaling were examined using wild type and bone-CKO mice tibia section A down-regulation in the pFGFR level, albeit slight, was observed (Figure **5B** **i**) in case of bone-CKO mice compared to wild type littermate controls. The downstream molecule of the RAS-MAP kinase pathway, pErk, was also found to be down-regulated in the bone-CKO mice compared to wild type littermate controls (n=3, Average intensity WT 10.5±0.57 vs. CKO 5.65±1.05) (Figure **5B** **ii**), further supporting the observation of reduced pFGFR levels. A 60% up-regulation in Sprouty 2 (n=3, Average intensity WT 8.5±2.3 vs. CKO 13.12±1.8) (Figure **5B** **iii**), a negative feedback inhibitor of the FGF-signaling pathway, was also observed. The concerted down-regulation of FGF signaling through MAP kinase pathways in the bone-CKO mice suggests that the deletion of POR has an effect upstream of FGF signaling. Decreased levels of pFGFR (Figure **6B**) as well pERK (Figure **6C**) were also observed in the POR knockdown 2T3 cell line (Figure **6A**), where POR was stably deleted using a shRNA lentivirus construct as described in our recent publication [32]. In this published work we showed that deletion of POR in both primary osteoblasts and osteoblast cells lines, MG63 and 2T3, decreased the Cx43 expression, which is regulated at the transcriptional level [32]. Cx43 expression and gap junction intercellular communication (GJIC) has been implicated in FGF signaling and ERK activation during chick limb development and mouse mesenchymal cells, as well as in osteoblast cell lines [50,51,52]. ERK mediated signaling can affect Runx2 and Osx, as ERK-mediated expression of both Runx2 and Osx has been shown in flavonoid Ugonin K-facilitated osteoblast differentiation and mineralization [53]. As mentioned previously, POR deletion will influence the activity of various CYPs and thus would alter the levels of various metabolites such as retinoic acid, cholesterol, fatty acids and steroids. Retinoic acid-mediated signaling is critical during embryonic development and retinoic acid is also a known teratogen. Retinoic acid has also been shown to coordinate FGF signaling during stem cell differentiation [54]. Estrogen, whose production is catalyzed by POR/CYP19, stimulated cultured rat anterior pituitary cell line (MtT/Se) growth by basic FGF (bFGF) and increased the expression of FGFR compared to cells without estrogen treatment [55]. Thus, many connections are known to exist between several POR metabolites and the FGF signaling pathway in other tissues or cell types, supporting our hypothesis of a POR effect on FGF in bone.

## Conclusion

**Figure 7:**
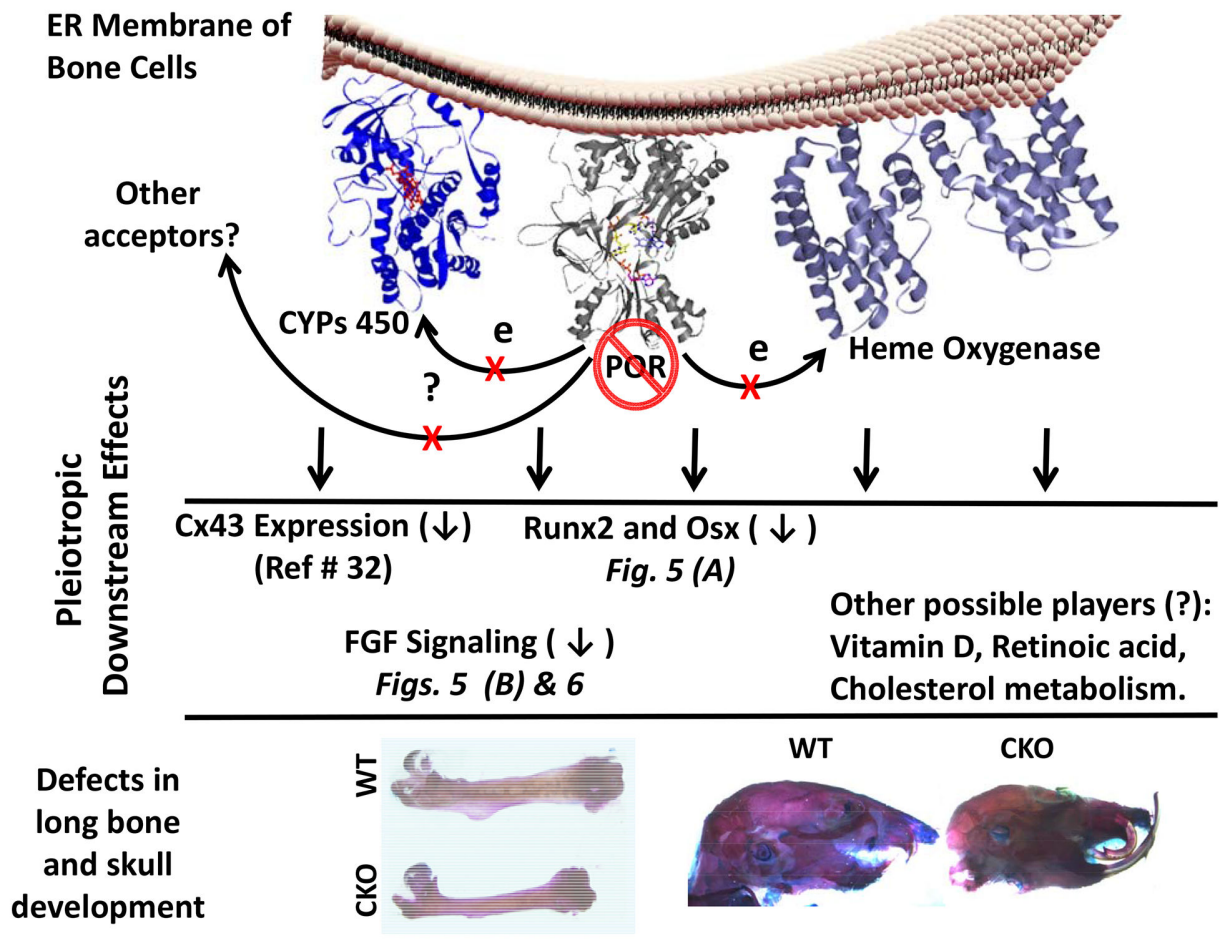
P450 Reductase and bone defects. P450 Oxidoreductase (POR), an endoplasmic resident redox protein, donates electrons to cytochromes P450 (CYPs450) and Heme oxygenase (HO). Deletion of POR in the bone cells affects CYPs450 and HO activity and inhibits the expression of Cx43, Runx2, Osx and FGF signaling. POR deficiency most likely also alters the concentration of CYP-dependent substrates or metabolites such as retinoic acid, vitamin D, and cholesterol, among others. These altered metabolic profiles along with Runx2, Osx and FGF signaling likely affect the bone and skull development as an outcome of POR deletion.

POR is the obligatory donor of electrons to the 50 microsomal cytochromes P450 and heme oxygenase, and it is also one of the known electron donors to other redox enzymes, such as squalene monoxygenase. Any mutation in POR that affects its activity or protein-protein interaction affinity will affect a wide variety of anabolic, catabolic, and developmental pathways, as the CYPs are involved in such diverse activities as drug and xenobiotic metabolism, steroid and cholesterol biosynthesis, production of second messenger molecules, bile acid synthesis, and formation and/or clearance of vasoactive compounds. To delineate the role of bone-expressed POR in skeletal development, we developed this animal model in an attempt to abrogate the detrimental systemic effects of the more global POR knockout and to provide an appropriate model to study the effects of the deficiency of a redox enzyme on embryonic development, specifically in bone. The abnormal skeletal development seen in human patients with POR mutations is most likely a culmination of defects in various pathways (Figure **7**). The targeted deletion of *POR* in the osteoprogenitor cells, using *DermoCre*, affected both body size and skeletal development. The aberrant development of the skull upon *POR* deletion in osteoprogenitor cells in this mouse model not only implicates POR in bone development but it recapitulates, at least partially, the observed craniofacial deformities and bone defects leading to fracture in severe POR-deficient human patients. The prior mouse model with conditional deletion of *Por* using *Prx1 Cre* failed to recapitulate the craniofacial deformity [27]. As the expression of POR in the liver was not affected in the bone conditional knockout of the *Por* gene in mice, it is of great interest to study the role of POR in bone and how it affects its development. The ability of cholesterol to partially rescue the observed phenotype in *Prx1CrePor* ^*lox/lox*^ mice, the role of abnormal retinoic acid metabolism and the down regulation of Cx43, Runx2, Osx and FGF signaling upon deletion of POR in bone cells, indicate that the pathway leading to defects in bone development might involve more gene products and possibly cross-talk between other signaling pathways and/or involvement of molecules such as vitamin D_3_ and estrogen. Though these pathways have been implicated in bone development, only direct relationships between POR deletion and Cx43 expression [32], hedgehog signaling [20,34], as well as FGFR signaling (present study) have been shown. Defects in bone development upon targeted deletion of POR in osteoprogenitor cells raise the possibility of localized effects of POR-dependent signaling pathways. This mouse model will also be useful in looking at the endocrine nature of bone in relation to POR-mediated formation of metabolites, as there is no evidence as yet that POR plays a physiological role independent of those of its redox partners.

## Supporting Information

Table S1 Table showing the frequency of different skull phenotype observed during examination of 20 CKO mice by naked eye. Mice were older than 3 weeks (this age was chosen as the skull deformities can be observed directly). The observed phenotypes were compared with sex-matched littermate controls. None of the wild type controls showed any skull deformities.
(DOCX)
