# Supplementary material for: Conditional Deletion of Cytochrome P450 Reductase in Osteoprogenitor Cells Affects Long Bone and Skull Development in Mice Recapitulating Antley-Bixler Syndrome: Role of a Redox Enzyme in Development
Source: PLoS One. 2013 Sep 25;8(9):e75638. doi: 10.1371/journal.pone.0075638 (PMC3783497; doi:10.1371/journal.pone.0075638)
Supplement: Table S1 — Table showing the frequency of different skull phenotype observed during examination of 20 CKO mice by naked eye. Mice were older than 3 weeks (this age was chosen as the skull deformities can be observed directly). The observed phenotypes were compared with sex-matched littermate controls. None of the wild type controls showed any skull deformities. (DOCX) [file pone.0075638.s001.docx]

Table S1

| **Phenotype** | **Mice #** |
| --- | --- |
| None observed | 6 |
| Tooth malocclusions | 1 |
| Dome-shaped skull | 7 |
| Tooth malocclusion and  dome-shaped skull | 6 |

Table showing the frequency of different skull phenotype observed during examination of 20 CKO mice by naked eye. Mice were older than 3 weeks (this age was chosen as the skull deformities can be observed directly). The observed phenotypes were compared with sex-matched littermate controls. None of the wild type controls showed any skull deformities.
